# Age at onset distinguishes clinical features and relapse risk in autoimmune glial fibrillary acidic protein astrocytopathy

**DOI:** 10.3389/fimmu.2026.1856124

**Published:** 2026-05-29

**Authors:** Jinbei Yu, Yunbo Shi, Xue Wang, Tianchun Wu

**Affiliations:** 1 The department of neurology, The First Affiliated Hospital of Zhengzhou University, Zhengzhou, China; 2 The department of Hepatobiliary and Pancreatic Surgery, The First Affiliated Hospital of Zhengzhou University, Zhengzhou, China

**Keywords:** age, autoimmune glial fibrillary acidic protein astrocytopathy, clinical manifestations, modified ranking scale, oligoclonal bands, relapse

## Abstract

**Background:**

Age is a critical factor influencing the clinical presentation of neuroimmunological diseases. This study aimed to investigate the different clinical characteristics of early-onset autoimmune glial fibrillary acidic protein astrocytopathy (EO-GFAP-A age < 45 years) and late-onset group(LO-GFAP-A age ≥ 45 years) (GFAP-A), and to preliminarily explore the correlation between age and relapse.

**Methods:**

We retrospectively enrolled 69 patients with first-onset GFAP-A who were admitted to the First Affiliated Hospital of Zhengzhou University from January 2020 to January 2025. All patients were followed-up for more than one year. We performed univariate analysis to compare clinical data between the two groups. Additionally, Kaplan-Meier survival analysis (with Log-rank test) and univariate Cox regression model were utilized to preliminarily assess the correlation between age and recurrence.

**Results:**

The proportions of patients with headache, fever, psychosis, and meningeal irritation signs were significantly higher in the EO-GFAP-A group than in the LO-GFAP-A group (*P* < *0.05*). Regarding imaging findings, involvement of the splenium of the corpus callosum was more frequent in the EO-GFAP-A compared to the LO-GFAP-A group (*P* < 0.05). Furthermore, the EO-GFAP-A group exhibited higher cerebrospinal fluid (CSF) nucleated white blood cell(WBC) counts and lower glucose levels than the LO-GFAP-A group (*P* < 0.05). Conversely, the LO-GFAP-A group showed a higher proportion of positive CSF oligoclonal bands (OCBs) and a higher one-year recurrence rate compared to the EO-GFAP-A group (*P* < 0.05). Kaplan-Meier survival curve demonstrated that the recurrence-free survival rate was significantly lower in the LO-GFAP-A group than in the EO-GFAP-A group (χ² = 7.8, *P* = 0.005). Univariate Cox regression analysis identified age as a significant predictor of recurrence risk. with each additional year of age associated with a 5.2% increase in the risk (HR = 1.052, 95% CI: 1.007–1.098, *P* = 0.022).

**Conclusion:**

Patients with EO-GFAP-A and LO-GFAP-A exhibit distinct clinical characteristics, and age is suggested to be a potential risk factor for relapse. Consequently, more aggressive therapeutic interventions and individualized long-term management strategies are warranted for LO-GFAP-A patients to mitigate the risk of disability.

## 1 Introduction

Autoimmune glial fibrillary acidic protein astrocytopathy (GFAP-A) is an autoimmune disease of the central nervous system(CNS) mediated by GFAP-IgG antibodies. The disease manifests a broad spectrum of clinical phenotypes, including meningitis, encephalitis, myelitis, or overlapping syndromes. Neuroimaging typically reveals a pathognomonic perivascular linear contrast enhancement (PLCT) (1–3). The proposed pathogenesis involves a breakdown of immune tolerance triggered by specific genetic backgrounds or external factor, such as tumors or infections. This breach permits autoreactive CD8^+^ T cells to cross the blood-brain barrier(BBB), where they recognize and attack GFAP-positive astrocytes (1, 4, 5). This immune response recruits macrophages and complement while releasing substantial inflammatory mediators, leading to astrocyte dysfunction and widespread CNS inflammation (1). Since its initial systematic description by Flanagan et al. in 2017, hundreds of cases have been documented worldwide (3). To date, most studies have concentrated on describing the clinical manifestations, pathological mechanisms, and treatment strategies of GFAP-A, primarily highlighting the overall disease profile. Previous studies have extensively explored autoimmune conditions such as neuromyelitis optica spectrum disorders (NMOSD), myelin oligodendrocyte glycoprotein antibody-associated disease (MOGAD), and multiple sclerosis (MS). These investigations have highlighted their distinct clinical profiles across different age groups. Late-onset neuroimmunological diseases (NMOSD, MOGAD, and MS) often present with worse recovery, aggressive disability progression, and distinct clinical features compared to younger-onset patients (6–11). There is a paucity of research focusing on the age-related clinical characteristics of GFAP-A (12, 13). The typical age onset age for GFAP-A generally falls between 40 and 50 years old (13). Accordingly, we stratified our clinical data using an age cut-off of 45 years. This study included 69 patients with GFAP-A who had a follow-up period exceeding one year. While GFAP-A typically responds favorably to immunotherapy, such as high-dose corticosteroids, relapses remain a clinical challenge. Given the paucity of data regarding relapse patterns (13, 14), we aimed to preliminarily explore the correlation between age and relapse to better understand the disease heterogeneity of the disease at different stages of onset. These findings aim to provide a scientific basis for individualized management, particularly for high-risk populations, thereby optimizing long-term treatment strategies.

## 2 Methods

### 2.1 Participants

This single-center study include a total of 69 patients hospitalized at the First Affiliated Hospital of Zhengzhou University from January 2020 to January 2025. All participants were experiencing their first onset of GFAP-A and had a minimum follow-up duration of one year. The diagnostic points of GFAP-A were based on previous literatures (2, 3), which include: (a) a definite diagnosis relies on the positive detection of GFAP-IgG antibodies in the cerebrospinal fluid(CSF), which demonstrates higher specificity than serum testing; (b) compatible clinical and radiological features, typically manifesting as meningoencephalitis, myelitis, or optic disc edema (or a combination thereof), with paraventricular linear radial enhancement on MRI (although not observed in all cases); and (c) the exclusion of other diagnoses. The inclusion criteria were as follows: (1) positive for GFAP-IgG in the CSF and/or serum by cell-based assay(CBA); (2) first onset with a follow-up period exceeding one year; (3) availability of complete clinical data, including CSF biochemical profiles tests, brain/spinal cord MRI findings. The exclusion criteria were: (1) coexistence of other CNS autoimmune antibodies (e.g. AQP4-IgG, MOG-IgG, autoimmune encephalitis-related antibodies, or paraneoplastic antibodies), excluding of non-neurological autoimmune diseases (e.g. rheumatic diseases). other neurological conditions causing deficits, such as cerebrovascular diseases; (2) incomplete imaging or laboratory data; or loss to follow-up. The detailed information of the cohort inclusion can be found in **Supplementary Figure 1**.

Serum and CSF samples were analyzed using an indirect immunofluorescence cell-based assay (CBA) with human embryonic kidney (HEK) 293 cells transfected with GFAP expression plasmids (Shanghai Genechem Co., Ltd., Shanghai, China). Samples exhibiting borderline antibody titers (defined as 1:10 in serum and 1:1 in CSF) were retested in duplicate by two independent readers to confirm reproducibility. Imaging findings were evaluated by two independent radiologists who were blinded to the clinical information. Inter-rater reliability was assessed using Cohen’s kappa (**Supplementary Table 1**). Furthermore, any ambiguous or borderline cases were assessed by two experienced neurologists to establish the final diagnosis.

### 2.2 Data collections

1)Baseline patient characteristics: age at onset (years), sex, and length of hospital stay (days); 2) Clinical manifestations: fever, headache, disturbance of consciousness, psychosis, cognitive disorder, extremity weakness, urinary and bowel dysfunction, paresthesia, tremor, dizziness, visual impairment, seizure, ataxia, meningeal irritation signs; 3)Biochemical findings: a. Initial CSF profiles: lumbar puncture pressure (mmH_2_O), white blood cell (WBC) counts (×10^6^/L), protein level (mg/L), glucose (mmol/L), chloride (mmol/L), lymphocyte percentage, IgG index, 24-hour IgG synthesis rate, CSF-specific oligoclonal bands (OCBs; types OCB II and III were considered positive), CSF viral infection (via antibody testing or high-throughput sequencing); b. Serum electrolytes: sodium and chloride levels (mmol/L); 4) Neuroimaging (Brain and spinal magnetic resonance imaging (MRI). Brain lesions locations: meninges, cortex/subcortex, periventricular/deep white matter, basal ganglia, corpus callosum, brainstem, cerebellum; Spinal cord leison locations: cervical, thoracic, and lumbar segments; 5) Tumor comorbidity (or Concurrent neoplasms).

Consistent with previous studies, the modified Rankin Scale (mRS) score was also employed to evaluate patients’ clinical functional status (15). The scale ranges from 0 (no symptoms) to 6 (death). mRS were assessed at admission, at the nadir of the disease, and at the final follow-up, respectively. Additionally, we evaluated disease relapse at 1 year and at the final follow-up.

## 3 Statistical analysis

All statistical analyses were carried out using SPSS version 27.0. Continuous data, summarized as median (interquartile range), were compared between the EO-GFAP-A group and LO-GFAP-A group using the independent samples t-test or Mann-Whitney U test, depending on the normality of distribution. Categorical data, presented as frequencies (percentage) were analyzed using the Chi-square test or Fisher’s exact test. The Kaplan-Meier method and Log-rank test were employed to plot recurrence curves and compare recurrence risks, respectively. Furthermore, Univariate Cox regression models were constructed to investigate the impact of age on relapse. *P* < 0.05 was considered statistically significant.

## 4 Results

### 4.1 Demographics of the study cohort

A total of 69 patients with confirmed GFAP-A who were followed up for more than 1 year were enrolled in this study. The cohort included 45 males and 24 females, with a male-to-female ratio of 1.88:1. The median age at onset was 44 years (range: 3–70 years), which is consistent with that reported in the literature (3). Concurrent tumors were identified in two patients (one with lung cancer and one with gallbladder cancer). The disease onset was predominantly acute or subacute, accounting for more than 85% of cases. There were 39 patients in the EO-GFAP-A group (≤ 45 years) and 30 patients in the LO-GFAP-A group (> 45 years). The median follow-up time was 31 months (range:12–60 months), with no statistically significant difference observed between the two groups (*P* = 0.735).

### 4.2 Comparisons of clinical characteristics in EO- GFAP-A and LO-GFAP-A

In terms of demographics, there was no statistical difference in sex distribution between the two groups. The mean age at onset was 27.9 ± 11.9 years in the EO-GFAP-A group and 58.8 ± 6.6 years in the LO-GFAP-A group. Significant differences in clinical manifestations were observed between the EO-GFAP-A and LO-GFAP-A groups (**Table 1**). The incidence of fever was higher in the EO-GFAP-A group than in the LO-GFAP-A group (89.7% vs. 50.0%, *P* < 0.001. Similarly, headache was more frequent in the EO-GFAP-A group (82.1% vs. 11.0%, *P* < 0.001. Coincidentally, meningeal irritation signs (MIS) were significantly more common in the EO-GFAP-A group (66.7% vs. 23.3%, *P* < 0.001. No significant differences were found in extremity weakness or disturbance of consciousness between the two groups. However, psychosis was significantly more prevalent in the EO-GFAP-A group (33.3% vs. 10.0%, *P* = 0.023). Although paresthesia and nausea occurred more frequently in the EO-GFAP-A group, these differences did not reach statistical significance (*P* = 0.058 and *P* = 0.064). Non-neurological autoantibody screening was performed in 33.3% of the patients, revealing an overall positivity rate of 34.8%. No significant difference in seropositivity was observed between the EO-GFAP-A and LO-GFAP-A groups (40.0% vs. 30.8%; *P* = 0.490).

**Table 1:** Demographics and clinical manifestations of the study cohort.

Demographics	EO-GFAP-A (n=39)	LO-GFAP-A (n=30)	*P-value*
Onset age (years)	27.92 ± 11.94	58.75 ± 6.56	0.000
Sex (male:female)	23:16	22:8	0.214
Admission time (d)	24.0 (22.25)	19.0 (12.50)	0.173
Follow-up time (m)	34.27 ± 15.45	30.92 ± 15.58	0.827
Clinical manifestations (%)			
Fever	35 (89.7)	15 (50.0)	0.000
Headache	32 (82.1)	11 (36.6)	0.000
urinary and bowel dysfunction	28 (71.8)	20 (66.7)	0.646
extremity weakness	31 (79.5)	24 (80.0)	0.958
cognitive disorder	19 (48.7)	10 (33.3)	0.199
disturbance of consciousness	21 (53.8)	10 (33.3)	0.089
psychosis	13 (33.3)	3 (10.0)	0.023
paresthesia	12 (30.8)	16 (53.3)	0.058
tremor	9 (23.1)	10 (33.3)	0.344
ataxia	6 (15.4)	4 (13.3)	0.810
dizziness	12 (30.8)	4 (13.3)	0.089
seizure	7 (17.9)	3 (10.0)	0.352
visual impairment	4 (10.3)	3 (10.0)	0.972
nausea and vomiting	16 (41.0)	6 (20.0)	0.063
meningeal irritation signs	26 (66.7)	7 (23.3)	0.000

### 4.3 Brain and spinal MRI imagings in EO- GFAP-A and LO-GFAP-A

**Figure 1:**
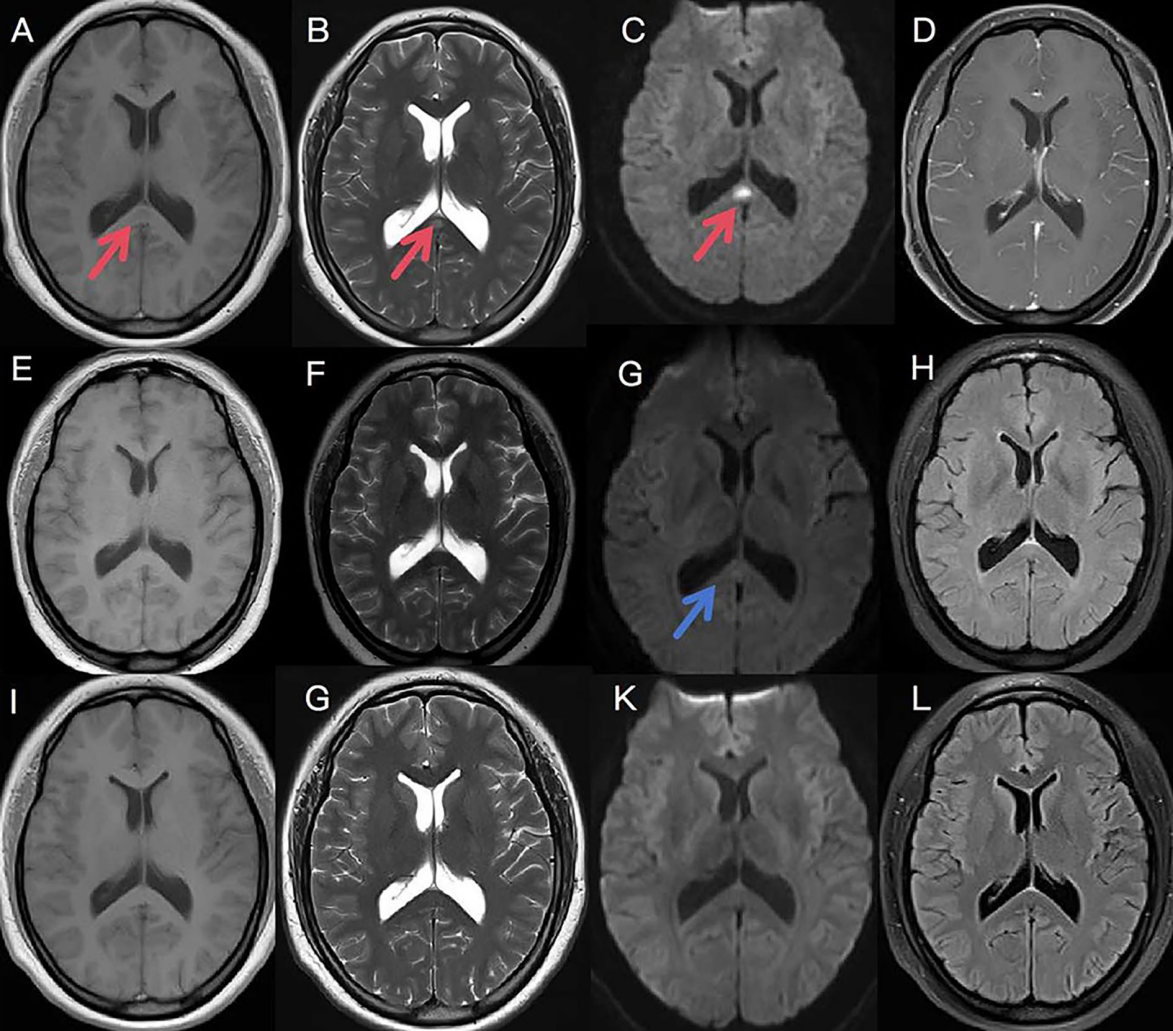
Representative MRI images of the splenium of the corpus callosum in a patient with GFAP-A. **(A-D)** Initial presentation at admission. Red arrows indicated lesions in the splenium of the corpus callosum.Brain MRI showed hypointensity on the T1-weighted image **(A)**, hyperintensity on T2-weighted **(B)** and diffusion-weighted imaging (DWI) MRI **(C)**, but no enhancement on the post-contrast T1-weighted image **(D)**. **(E-H)** Follow-up images approximately 2 weeks after initiation of mmunotherapy. Near-complete resolution of the lesion was observed on T1 **(E)**, T2 **(F)**, and post-contrast T1 **(H)** sequences.A decreased signal intensity is noted on DWI **(G)** (blue arrow). **(I-L)** Follow-up images at about 5 months after discharge, demonstrating complete resolution of the lesion.

Comparisons of neuroimaging findings between the two groups are presented in **Table 2**. Intracranial lesions were widely distributed throughout the brain. In addition to the typical imaging features previously described, a subset of patients exhibited lesions involving the splenium of the corpus callosum (**Figure 1**). Notably, the frequency of corpus callosum lesions was significantly higher in the EO-GFAP-A group than that in the LO-GFAP-A group (*P =* 0.012). Meninges involvement was more frequent in the EO-GFAP-A group, although the difference did not reach statistical significance (*P =* 0.076). Spinal cord involvement was most commonly observed in the cervical and thoracic segments. However, no significant differences were found in the distribution of spinal cord lesions between the two groups.

**Table 2:** Brain and spinal MRI imagings in EO- GFAP-A and LO-GFAP-A.

Neuroimaging findings(%)	EO-GFAP-A (n=39)	LO-GFAP-A (n=30)	*P-value*
meninges	14 (35.9)	5 (16.7)	0.076
cortex/subcortex	9 (23.1)	4 (13.3)	0.305
periventricular/deep white matter	9 (23.1)	4 (13.3)	0.305
basal ganglia	15 (38.5)	6 (20.0)	0.098
corpus callosum	10 (25.6)	1 (3.3)	0.012
cerebellum	3 (7.7)	3 (10.0)	0.736
brainstem	8 (20.5)	7 (23.3)	0.778
cervical spinal cord	20 (51.3)	19 (63.3)	0.317
thoracic spinal cord	22 (56.4)	20 (66.7)	0.387
lumbar spinal cord	6 (15.4)	4 (13.3)	0.810

### 4.4 Serum and cerebrospinal fluid findings in EO- GFAP-A and LO-GFAP-A

CSF findings are summarized in **Table 3**, The EO-GFAP-A group exhibited significantly higher CSF pressure compared to the LO-GFAP-A group (198.08 ± 64.20 vs 147.92 ± 61.64, *P* = 0.021). CSF WBC counts were markedly elevated in the EO-GFAP-A group (113.0 vs 49.5 P = 0.014). However, the percentage of CSF lymphocytes showed no difference (*P* = 0.566). CSF glucose levels were substantially lower in the EO-GFAP-A group (median: 2.64 vs 3.19 P = 0.025). The proportion of positive CSF viral tests was comparable between the two groups (*P* = 0.648). No significant differences were observed in the IgG index (*P* = 0.451) or the 24-hour IgG synthesis rate (*P* = 0.490). OCBs refers to two or more distinct, discrete, and heterogeneous narrow protein bands appearing in the gamma-globulin region near the cathodic end. They reflect local B-cell clonal proliferation and intrathecal IgG synthesis (16, 17).CSF OCBs are categorized into four patterns, with types 2 and 3 considered positive for specific intrathecal synthesis. The proportion of patients with positive OCBs was higher in the LO-GFAP-A group (35.3% vs 70.4%, *P* = 0.006). Furthermore, no appreciable differences were found in serum sodium (136.0 vs 136.0, *P* = 0.628), serum chloride (98.41 ± 6.17 vs 99.58 ± 5.17, *P* = 0.650), or the proportion of hyponatremia between the two groups.

**Table 3:** Serum and CSF findings in EO- GFAP-A and LO-GFAP-A.

CSF findings	EO-GFAP-A (n=39)	LO-GFAP-A (n=30)	*P-value*
CSF pressure(mmH2O)	198.08 ± 64.20	147.92 ± 61.64	0.021
Cell count(×10^6^/L)	113.0 (156.0)	49.5 (108.75)	0.014
Protein level(mg/L)	965.3 (983.73)	833.75 (563.33)	0.771
Glucose (mmol/L)	2.64 (0.96)	3.19 (1.03)	0.025
Chloride(mmol/L)	118.22 ± 7.52	120.04 ± 7.71	0.213
Lymphocyte ratio(%)	85 (11.7)	86 (11.5)	0.566
CSF viral infection (%)	11 (28.2)	7 (23.3)	0.648
IgG production index	0.76 ± 0.22	0.79 ± 0.31	0.451
24-hour IgG synthesis rate(mg/24h)	17.34 ± 15.84	20.54 ± 23.40	0.490
OCB (%)	12 (35.3)	19 (70.4)	0.006
Serum findings			
Serum sodium(mmol/L)	136.0 (12.0)	136.0 (5.25)	0.628
Hyponatremia level(mmol/L)	125.9 (10.5)	131.0 (8.6)	0.101
Hyponatremia (%)	20 (51.3)	20 (40.0)	0.352
Serum chloride(mmol/L)	98.41 ± 6.17	99.58 ± 5.17	0.650

CSF, cerebral spinal fluid; OCB, oligoclonal bands.

### 4.5 Analysis of relapse factors of GFAP-A

**Figure 2:**
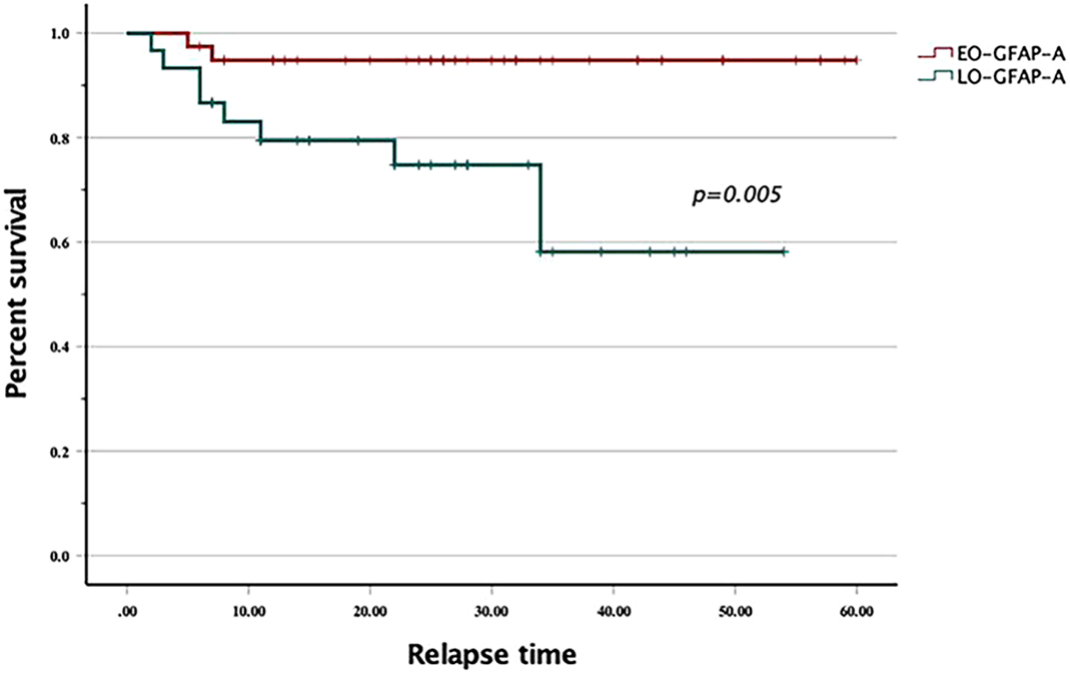
Time to relapse in the EO-GFAP-A and LO-GFAP-A groups. LO-GFAP-A patients exhibited a significantly shorter time compared to those in the EO-GFAP-A group (*P =* 0.005).

The follow-up duration between the two groups were comparable. Both groups demonstrated favorable responses to acute-phase treatment, with the majority of patients achieving significant symptomatic improvement. In this study, glucocorticoids were administered to 66 patients (95.7%), and intravenous immunoglobulin (IVIG) was utilized in 29 patients (42.0%). Following disease onset, 12 patients (17.6%) received immunosuppressant therapy, which primarily consisted of mycophenolate mofetil (n=6), efgartigimod (n=4), rituximab (n=1), and ofatumumab (n=1). No statistically significant difference was observed in the administration of immunosuppressants between the EO-GFAP-A and LO-GFAP-A groups (17.9% vs. 16.7%, *P* = 0.889). The median modified Rankin Scale (mRS) score at admission was 2.0 in the EO-GFAP-A group and 3.0 in the LO-GFAP-A group (**Table 4**). At the nadir of the disease, the median mRS score was 4.0 in both groups. By the last follow-up, the median mRS scores were 1.0 in the EO-GFAP-A group and 2.0 in the LO-GFAP-A group, with no notable difference between the two groups (*P* = 0.580). A total of 9 patients experienced relapse during the 1-year follow-up, with the LO-GFAP-A group exhibiting a markedly higher recurrence rate (*P* = 0.035). At the final follow-up, the overall recurrence rate remained significantly higher in the LO-GFAP-A group compared to the EO-GFAP-A group (*P* = 0.007). The mean time to relapse was 7 months (range: 2–34 months). Survival analysis using the Kaplan-Meier method demonstrated that the recurrence-free survival rate was notably lower in the LO-GFAP-A group than in the EO-GFAP-A group. The Log-rank test confirmed a robust difference between the survival curves (χ²=7.8, *P* = 0.005) (**Figure 2**). Furthermore, univariate Cox regression analysis identified age at onset as a significant predictor of relapse (HR = 1.052, 95% CI: 1.007–1.098, *P* = 0.022); for every 1-year increase in age at onset, the risk of recurrence increased by 5.2%.

**Table 4:** mRS and Relapses in EO- GFAP-A and LO-GFAP-A.

Varients	EO-GFAP-A (n=39)	LO-GFAP-A (n=30)	*p-value*
mRS			
mRS (admission)^*^	2.0 (1-5)	3.0 (1-5)	0.165
mRS (nadir)^*^	4.0 (1-5)	4.0 (1-5)	0.747
mRS (final follow-up)^*^	1.0 (0-6)	2.0 (0-6)	0.580
Follow-up time(m)^#^			
	34.27 ± 15.45	30.92 ± 15.58	0.827
Relapse			
Relapse rate at 1 year (%)	2 (5.1)	7 (23.3)	0.035
Number of relapse^*^	1.0 (1-1)	1.0 (1-3)	0.272
Relapse rate at final follow-up (%)	2 (5.1)	9 (30.0)	0.007
Relapse time (m)^*^	6 (5-7)	12.8 (2-34)	0.476

* median(range); **^#^**mean ± SD.

## 5 Discussion

### 5.1 Principal findings

This study systematically characterizes the differences in clinical phenotype, imaging features, CSF indicators, prognosis, and recurrence between early-onset (< 45 years) and late-onset (≥ 45 years) groups through a stratified analysis of 69 patients with GFAP-A. Our findings indicate that age at onset significantly modifies the clinical expression of GFAP-A. The EO-GFAP-A group exhibited a prominent inflammatory phenotype, characterized by higher frequencies of headache, fever, and meningeal irritation signs. Furthermore, this group demonstrated significantly elevated CSF cell counts, reduced glucose levels, and a greater susceptibility to corpus callosum involvement. Conversely, the LO-GFAP-A group was characterized by prominent intrathecal antibody synthesis, reflected by a higher OCB positivity rate and a significantly elevated risk of disease recurrence.

### 5.2 Interpretation of the main research results and comparison with previous literatures

In this study, the EO-GFAP-A group exhibited more prominent inflammatory symptoms, consistent with the core clinical manifestations of GFAP-A (fever, headache, disturbance of consciousness, and seizures) (12). Early-onset patients had significantly higher CSF WBC counts and lower glucose levels, suggesting a more pronounced CNS inflammatory response in younger patients. This phenomenon may be attributed to age-associated characteristics of immune reactivity. Younger individuals typically possess more robust immune systems. Consequently, they may mount stronger autoimmune responses against self-antigens. This manifests as more overt clinical symptoms and CSF inflammatory changes. Guo et al. demonstrated that GFAP-A is characterized by prominent lymphocytic infiltration, specifically, the perivascular accumulation of CD8^+^ cytotoxic T cells targeting astrocytes alongside complement deposition (5). This indicates that the disease is driven by a T-cell-mediated targeted immune response, resulting in an acute inflammatory reaction rather than chronic immune activation. Given that younger individuals generally possess a more active T-lymphocyte repertoire, this may account for their heightened inflammatory presentation. However, the specific mechanisms warrant further investigation. This hypothesis is further supported by a recent pediatric study, which found that CSF pleocytosis to be highly prevalent, with a median cell count reaching 245 cells/μL (18), significantly exceeding levels reported in many adult studies (3, 19). The findings further corroborate the notion that EO-GFAP-A patients exhibit more pronounced CNS inflammatory responses. The influence of age on disease phenotype is also observed in other neuroimmunological disorders. The pathological hallmark of NMOSD is AQP4-IgG-mediated complement activation, resulting in focal astrocytic necrosis (20). In patients with NMOSD, early-onset cases are more prone to optic neuritis, whereas late-onset patients are more likely to present with spinal cord involvement and experience greater disability; these differences may be associated with immunosenescence and the functional decline of the BBB (6). In contrast, the pathology of MOGAD is primarily characterized by antibody-dependent cellular phagocytosis with relative preservation of astrocytes (21, 22). Late-onset patients tend to have fewer relapses but face a higher risk of disability accumulation, which may be linked to immunosenescence and diminished B-cell function (9, 23). MS is predominantly a T-cell-mediated demyelinating disease, with B cells and antibodies playing a role in a subset of patients or at specific stages of the disease (24). A review by Capasso et al. (2023) elucidated the pathological differences between early- and late-onset MS from the perspective of aging, noting that early-onset patients are typically dominated by inflammatory responses, while neurodegeneration is relatively more prominent in late-onset cases (11). The fundamental mechanisms underlying age-related phenotypic variations across diverse demyelinating diseases remain elusive (23), though distinct pathogenic processes may contribute to this phenomenon.

The imaging manifestations of GFAP-A are diverse, with commonly affected sites including the cortex and subcortical white matter, periventricular white matter, basal ganglia, thalamus, brainstem and cerebellum, meninges, spinal cord, and optic nerve. The most characteristic neuroimaging feature is the hallmark perivascular linear contrast enhancement (1, 12, 25). Involvement of the splenium of the corpus callosum is relatively uncommon (approximately 5%) and may present as reversible splenial lesion syndrome (RESLES) (26–29). However, in our study, corpus callosum involvement was markedly higher (approximately 15.9%). Given that lesions in this region are highly sensitive to corticosteroids and can resolve completely after treatment, this finding may be attributable to our inclusion of treatment-naïve patients at admission. Furthermore, the proportion of splenial involvement was significantly higher in the EO-GFAP-A group (25.6%) than in the LO-GFAP-A group (3.3%). Previous studies have indicated that the splenium of the corpus callosum possesses a unique vascular supply structure that predisposes it to BBB breakdown, intramyelinic edema, and tissue damage (30, 31). Philipp et al. (32) found no evidence of local glucose hypometabolism in RESLES using positron emission tomography/computed tomography(PET/CT). It furtherly confirmed that RESLES represents intercellular, intramyelinic oedema rather than typical intracellular cytotoxic oedema (32). GFAP, as the cytoskeletal protein of astrocytes, plays a critical role in the interaction between astrocytic end-feet and blood vessels, thereby maintaining BBB stability (33, 34). This interaction may exhibit age-related differences, potentially rendering the splenium of the corpus callosum more susceptible to reversible edema and demyelination under systemic immune attack in younger patients. Additionally, the higher prevalence of psychosis in EO-GFAP-A group may also be related to corpus callosum involvement.

Recent studies have indicated that a substantial proportion of patients with GFAP-A have pre-existing autoimmune diseases (3, 26, 35, 36). These findings suggest that GFAP-A is not an isolated form of encephalitis but is significantly associated with systemic rheumatic diseases, such as Sjögren’s syndrome, systemic lupus erythematosus, and rheumatoid arthritis. While such comorbidities generally do not impact the efficacy of acute-phase corticosteroid therapy, careful consideration is warranted when selecting long-term immunosuppressive regimens. Therefore, we recommend that all patients with GFAP-A undergo comprehensive screening for systemic non-neurological autoantibodies.

GFAP-A generally exhibits a favorable response to immunomodulatory therapy. First-line treatment primarily consists of high-dose corticosteroids and IVIG, with most patients responding well and following a predominantly monophasic disease course (12, 19, 26). Our study found that the median mRS score at the last follow-up of the patients was 2 (range: 0-6), consistent with previous reports. However, disease recurrence is not uncommon. Recent research indicates an overall recurrence rate of approximately 14%-30% (13, 14, 37). In our cohort, the relapse rate at the last follow-up was 15.9%, and the overall 1-year relapse rate was 13.0%. These variations may be attributed to differences in study population, follow-up durations, and racial backgrounds. Notably, we found that the relapse rate in the LO-GFAP-A group was significantly higher than that in the EO-GFAP-A group. Regarding risk factors for relapse, a recent nationwide French cohort study identified concurrent tumors as a significant factor increasing recurrence risk (13), and OCBs positivity has also been implicated (14). Furthermore, Kimura et al. found that older age was positively associated with unfavorable outcomes (36). Our findings provide the first evidence of a potential association between age and relapse risk, with the risk increasing by 5.2% for each additional year of age. Additionally, we observed a higher OCBs positivity rate in the late-onset group. CSF GFAP antibodies serve as the diagnostic gold standard for GFAP-A (3). GFAP antibodies-mediated immune attacks lead to astrocyte dysfunction and white matter damage (38). OCBs reflect a continuous, chronic CNS immune inflammatory process. The presence of OCBs indicates ongoing intrathecal immunoglobulin synthesis, which is associated with a chronic disease course and recurrence risk in various autoimmune diseases (39). In this study, more than half of the patients were OCB-positive, aligning with previous findings (12, 14, 40). The higher OCBs positivity in late-onset patients suggests humora more prominent intrathecal humoral immune response in LO-GFAP-A group. We hypothesize that with advancing age, the risk of immune dysregulation and the expansion of autoreactive B-cell clones increases. This persistent humoral immune activation is closely linked to relapse. Given the distinct clinical characteristics associated with different ages of onset, treatment strategies should be tailored accordingly. For instance, in NMOSD, early-onset patients have been shown to respond better to acute-phase therapy; and early initiation of rituximab is recommended for this subgroup (41, 42). Consequently, there remains a significant unmet clinical need for individualized treatment strategies in the elderly GFAP-A population. For late-onset patients, more aggressive therapeutic strategies should be adopted to prevent relapse, warranting further investigation.

### 5.3 Strengths and conclusion

In conclusion, this study represents the first age-stratified comparative investigation into the distinct clinical characteristics of EO-GFAP-A and LO-GFAP-A, providing further insights into the nature of the disease. The EO-GFAP-A group was characterized predominantly by inflammatory manifestations, such as headache and fever, along with more frequent involvement of the splenium of the corpus callosum. Conversely, LO-GFAP-A group exhibited more prominent chronic persistent humoral immune responses and significantly higher relapse rates. Consequently, the adoption of more aggressive therapeutic interventions and individualized long-term management strategies is warranted for patients in the LO-GFAP-A group.

### 5.4 Limitations

This study has several limitations. First, its retrospective, single-center design, with all patients recruited from a single institution, may introduce selection bias. Second, the limited sample size precluded the performance of multivariate COX regression analysis. Future multicenter studies with larger cohorts are warranted to further elucidate the factors associated with relapses.

## Data Availability

The original contributions presented in the study are included in the article/**Supplementary Material**. Further inquiries can be directed to the corresponding author.

## Supplementary material

Supplementary Figure 1 Study flow diagram of patient selcetion.

Supplementary Table 1 Inter-rater Reliability (Cohen’s κ) for Neuroradiological Assessments.
