# Supplementary figures and images for: Age at onset distinguishes clinical features and relapse risk in autoimmune glial fibrillary acidic protein astrocytopathy
Source: Front Immunol. 2026 May 29;17:1856124. doi: 10.3389/fimmu.2026.1856124 (PMC13259700; doi:10.3389/fimmu.2026.1856124)

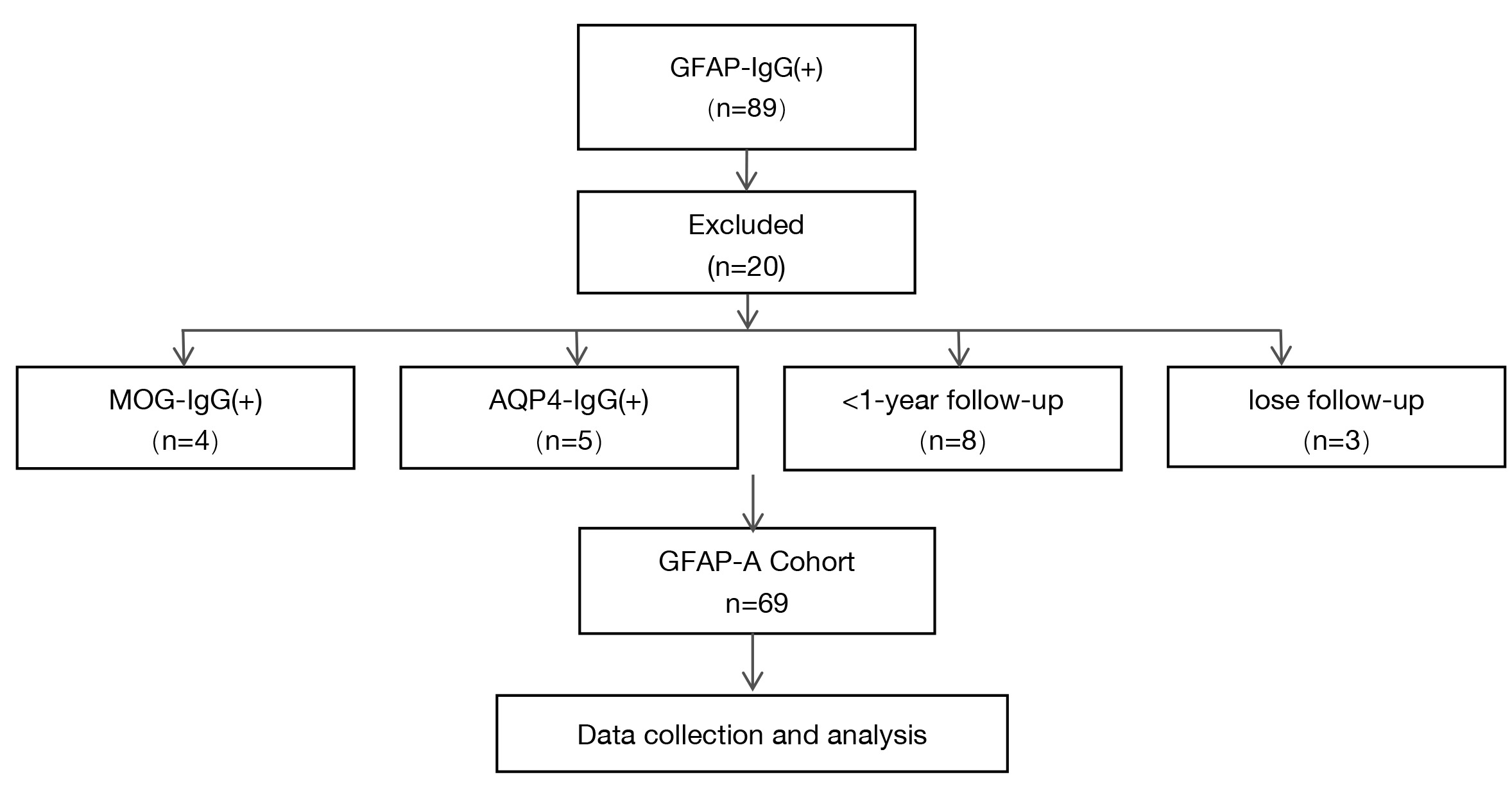

Supplement: Supplementary Figure 1 — Study flow diagram of patient selcetion. [file Image1.jpeg]
