# Supplementary material for: Age at onset distinguishes clinical features and relapse risk in autoimmune glial fibrillary acidic protein astrocytopathy
Source: Front Immunol. 2026 May 29;17:1856124. doi: 10.3389/fimmu.2026.1856124 (PMC13259700; doi:10.3389/fimmu.2026.1856124)
Supplement: Supplementary Table 1 — Inter-rater Reliability (Cohen’s κ) for Neuroradiological Assessments. [file Table1.docx]

Table S1 Inter-rater Reliability (Cohen’s κ) for Neuroradiological Assessments

| Neuroimaging findings | Kappa  κ | 95% CI | *P-value* |
| --- | --- | --- | --- |
| meninges | 0.802 | 0.630-0.935 | ＜0.001 |
| cortex/subcortex | 0.794 | 0.593-0.951 | ＜0.001 |
| periventricular/deep white matter | 0.830 | 0.634-0.960 | ＜0.001 |
| basal ganglia | 0.775 | 0.609-0.933 | ＜0.001 |
| corpus callosum | 0.854 | 0.641-1.000 | ＜0.001 |
| cerebellum | 0.841 | 0.574-1.000 | ＜0.001 |
| brainstem | 0.881 | 0.725-1.000 | ＜0.001 |
| cervical spinal cord | 0.884 | 0.741-0.971 | ＜0.001 |
| thoracic spinal cord | 0.880 | 0.757-0.971 | ＜0.001 |
| lumbar spinal cord | 0.832 | 0.609-1.000 | ＜0.001 |
